# Behavioral intervention for sleep problems in childhood: a Brazilian randomized controlled trial

**DOI:** 10.1186/s41155-019-0118-3

**Published:** 2019-01-28

**Authors:** Renatha El Rafihi-Ferreira, Maria Laura Nogueira Pires, Edwiges Ferreira de Mattos Silvares

**Affiliations:** 1 Universidade de São Paulo. Avenida. Prof. Mello Moraes, 1721. Butantã, São Paulo, SP 05508030 Brazil; 2 Universidade Estadual Paulista “Julio de Mesquita Filho”, Faculdade de Ciências e Letras de Assis, Avenida Dom Antônio 2100. Parque Universitário, Assis, SP 19806900 Brazil

**Keywords:** Behavioral intervention, Sleep problems, Behavior, Children, Sleep

## Abstract

**Background:**

Sleep problems are common in children and can have an effect on behavioral and emotional functioning. Despite the importance of sleep for children’s health, there is a lack of studies on this topic in Brazil. The aim of this study was to evaluate the efficacy of behavioral intervention for sleep problems in young children and to investigate the effects on their daytime behavior.

**Methods:**

Sixty-two children (ages 1–5; M = 2.3, SD = 1.3) with bedtime problems and night waking were randomized to a parent-based intervention or a wait list control group. After the waiting period, the wait list participants were offered treatment. The intervention was composed of five sessions over 2 months, during which the parents were educated on their child’s sleep and received guidance on the establishment of sleeping schedules and routines and on the use of extinction and positive reinforcement techniques. Sleep patterns and behavior problems were assessed with parent-report measures. Sleep patterns were also assessed with actigraphy. Assessments were completed at pre-intervention, post-intervention, 1-month follow-up, and 6-month follow-up.

**Results:**

Children who received intervention showed greater baseline to post-treatment improvements in sleep latency, night waking, behavior such as resisting going to bed, the desire to sleep with their parents, and daytime behavior than the control group. These improvements were maintained at follow-up.

**Conclusions:**

We concluded that behavioral parent-based intervention is effective in improving the quality of sleep and the diurnal behavior of children. This study provides initial support for use of this protocol in psychology clinics/schools. Brazilian clinical trials registration, RBR-4kxxd5. Retrospectively registered on December 13, 2016.

## Background

Sleep problems such as bedtime problems and night waking are quite common, with a prevalence of between 20% and 30% in infants, toddlers, and preschool-aged children (Mindell & Moore, 2014; Mindell, Kuhn, Lewin, Meltzer, & Sadeh, 2006). Prevalence in Brazil is in line with international records. A study developed by Pires, Vilela, and Câmara (2012) shows that one in two children has difficulty falling asleep and one in three awakens several times during the night and is sleepy during the day.

Sleeping difficulties are associated with child behavioral and emotional problems (Meltzer, 2010; Moore, 2010). Several studies (Blunden & Chervin, 2008; Blunden & Chervin, 2010; Byars, Yeomans-Maldonado, & Noll, 2011; Cortesi, Giannotti & Ottaviano, 1999; Hall, Zubrick, Silburn, Parsons, & Kurinczuk, 2007; Rafihi-Ferreira, Silvares, Pires, Assumpção-Jr, & Moura, 2016; Scher, Zukerman, & Epstein, 2005; Stein, Mendelsohn, Obermeyer, Amromin, & Benca, 2001), in which the association between sleep quality and behavioral problems was investigated, demonstrated a relationship between externalizing and internalizing problems and sleep problems in children. This association, as concerning as it is, becomes even more serious when considering that childhood sleep problems may persist and that they constitute a greater risk for other behavioral problems. These empirical relationships, already identified in certain longitudinal studies (Gregory, Ende, Willis, & Verhulst, 2008; Hall et al., 2007; Scher et al., 2005), indicate the importance of intervening as soon as possible in order to prevent the persistence of sleep problems over the years from becoming strongly associated with other behavioral problems such as anxiety, depression, and aggression.

Behavioral treatment approaches for children with bedtime problems and night waking are supported by the literature, which should be the first therapeutic choice according to the American Sleep Academy (Thomas, Moore, & Mindell, 2014). The empirically supported interventions in the context of sleeping problems in children include behavioral treatments implemented primarily by parents, with information on establishing pre-sleep routines, positive reinforcement, and extinction (Mindell et al., 2006).

There are a number of findings on the effectiveness of behavioral approaches in the treatment of childhood sleep problems. For example, a randomized controlled clinical study by Mindell, Telofski, Wiegand, and Kurtz (2009) assessed the impact of establishing a sleep routine on maternal sleep and mood in 405 seven-month-old children and their mothers. The results showed a decrease both in sleep onset latency and in night waking, thus indicating an improvement in infant sleep. An improvement in maternal mood was also observed. Another randomized clinical trial (Mindell et al., 2011a) with 264 mothers and their children aged 6 months to 3 years assessed the effectiveness of an internet-based intervention for childhood sleep problems. In this study, there were three study conditions: a bedtime routine condition, a bedtime routine + behavioral internet-based condition (the Customized Sleep Profile), and a wait list control. The guidelines in this study involved the following: establishing routines and breaking inappropriate associations with sleep onset. While in one of the groups, the establishment of routines was informed in a detailed manner for the caregivers, with specific guidelines such as bath, moisturizing massage lotion, and calm activities such as a lullaby and reducing the light around 30 min at the end of the bath. The results showed that in both treatment groups, significant improvements were observed in sleep onset latency and the number/duration of night wakings. An increase in total sleep time was observed, in addition to the confidence of the mothers in the management of their children’s sleep. The improvements were observed in the first week, with additional benefits in the second week. There were also improvements in maternal variables, both in terms of sleep and in the mood as well.

Review studies involving a series of investigations on treatment have also shown similar results. The review conducted by Mindell et al. (2006), for example, identified the efficacy of behavioral treatments for bedtime problems and frequent night wakings in infants. The authors reported a finding that most of the children submitted to the intervention experienced improved sleeping (in 80% of the studies reviewed). Moreover, the positive results were maintained for 3 to 6 months after completing treatment. A systematic review based on the meta-analysis (Meltzer & Mindell, 2014) demonstrated a moderate level of evidence for the behavioral treatment for insomnia in infants and toddlers. Another review study conducted by an advisory group (Morgenthaler et al., 2006) of the American Academy of Sleep Medicine had identified that behavioral interventions, such as extinction techniques, establishment of routines, preventive education for parents, and sleep hygiene habits should be considered to be effective therapies for problems related to lying down and awakening at night, resulting in an improvement in sleep patterns. Likewise, scholars (Moore, 2010; Tikotzky & Sadeh, 2010) have already indicated that behavioral interventions, administered by the parents, were effective in the short- and long-term for the management of insomnia in children.

It is important to add that treatment for childhood insomnia and good sleep quality may be beneficial for improving the child’s learning, aggression, mood, and behavior (Mindell et al., 2006). On the other hand, it is necessary to recognize that the literature (Mindell et al., 2006; Morgenthaler et al., 2006; Schlarb, Velten-Schurian, Poets, & Hautzinger, 2011) indicates the need for research that assesses the impact of the intervention on a child’s behavior and mood, as well as the use of objective measures (such as actigraphs) for the evaluation of sleep patterns.

Although sleep-related behavioral problems in children are receiving increasing importance worldwide, we recently reviewed RCTs of intervention for sleep in children and found no studies performed in Brazil (Rafihi-Ferreira, Pires, & Silvares, 2014). Given the importance of interventions in the reduction and prevention of health risks in children, the present study has two aims: (1) to evaluate the efficacy of behavioral intervention for childhood insomnia through a program prepared for Brazilian parents and (2) to investigate the effect on their children’s daytime behavior. Our first hypothesis was that the behavioral intervention resulted in the children’s improved sleep, characterized by reduction in their sleep latency, fewer night wakings, improvement in behavior such as resisting going to bed, and the desire to sleep with their parents, being the primary outcome. The second hypothesis was that the intervention could also result in children’s improved daytime behavior, being the secondary outcomes.

## Method

### Design and ethics

The study had two phases. In the first phase, we compared the intervention group with the wait list control group. In the second phase, the wait list group received the intervention and was reassessed.

The study employed a two-arm, randomized, and controlled trial design. The participants were randomized between intervention and control groups (waiting list). Outcomes were assessed at post-treatment and 1- and 6-month follow-up.

This study was approved by the Research Ethics Committee of the universities where the services were performed and was registered under Brazilian clinical trials registration (RBR-4kxxd5), http://www.ensaiosclinicos.gov.br/rg/RBR-4kxxd5/.

### Participants, recruitment, screening, and randomization

The recruitment of the participants occurred following interviews given by the first author about childhood sleeping problems on the radio, in newspapers, and on TV. The study’s assessments and interventions were conducted at the school clinics of the Instituto de Psicologia of Universidade de São Paulo (USP); the Universidade Estadual Paulista “Júlio de Mesquita Filho” (UNESP; CPPA—Centro de Pesquisa e Psicologia Aplicada “Dra Betti Katzenstein”), Assis, SP; and the Universidade Estadual de Londrina (UEL). Interested families made contact via telephone, were informed about the purpose of the study, and queried about their eligibility.

During the participant selection process, the following inclusion criteria were defined: (1) Parents/caregivers of children, 1 to 5 years of age who presented sleep-related behavioral problems (insomnia, due to sleep onset latency, association type, insomnia due to limit-setting difficulties, and a combination of the two). (2) A child who has any of the following characteristics listed below at a frequency of at least three times a week: (1) it takes about 30 min or more to fall asleep, (2) resists and/or protests going to bed, (3) night wakings, and (4) only sleeps in the presence of parents.

The study exclusion criteria were as follows: children with neurological impairment, those having a psychiatric diagnosis, those having sleep problems resulting from physiological conditions, and those whose caregivers could not attend the in-person sessions. The exclusion criteria were applied before the randomized assignment to the groups.

The individuals who met the inclusion criteria mentioned above were numbered by order of arrival and randomized, from blocks of eight participants, to the control (wait list) and intervention groups. The participants received no compensation for their participation in the study.

### Sample size

Based on previous work (Wiggs & Stores, 1998), the sample size in each group to detect a mean difference of 3.33 points on Composite Sleep Disturbance Score (CSDS) with a power of 95% is 20. The sample size for each group was then increased to 30 to account for dropouts or losses in follow-up. Based on this estimate, calls were accepted from those interested in participating in the study up to 1 month after the recruitment announcements began.

### Procedure

#### Intervention

##### Behavioral intervention for sleep problems in childhood

The intervention program consisted of five sessions lasting 60 min, with the first three being performed weekly and the last two fortnightly. The sessions were one-on-one with the mothers of the children and were conducted by the first author of the study who has a bachelor’s degree and a master’s degree in behavioral psychology and at the time was a doctoral student in clinical psychology. The researcher received weekly supervision from her supervisors/professors with extensive experience in child behavioral therapy and applied psychology in sleep problems. The intervention program used in this study was based on the Durand (2008).

The initial focus of the program was for caregivers to understand the role of the environment, i.e., parental behavior in terms of their children’s complaints. Later, it was established that the teaching of appropriate behavior to be practiced by the parents when putting the child to sleep and during night wakings would result in (1) reduction in the child’s problematic behavior and (2) an increase in the frequency of appropriate behavior. The intervention occurred by way of parental guidance with explanations regarding the maintenance of the child’s behavior and guidance regarding sleep-related behavior, including sleep hygiene; establishment of pre-sleep routine with calm activities that end in the bedroom, so that the child associates such relaxing activities with the time to go to sleep; extinction to reduce inadequate responses and positive reinforcement to increase appropriate sleep behavior. Table 1 below presents the intervention program session by session, with the topics covered in each session.

**Table 1:** Objectives and topics covered in the intervention program

Sessions	Topics covered
Session 1	1. Functional analysis based on the records in the sleep diaries: understanding the maintenance of sleep-related behavior-problem 2. Establish target behavioral changes: (1) extinction for the behaviors that compete with the self-accommodation, which are necessary for the process of falling asleep and (2) reinforcement of the behaviors that favor falling asleep 3. The child’s sleep, sleep hygiene, and the importance of establishing the pre-sleep routine
Session 2	1. Review of the sleep diaries 2. Strengthen the importance of the pre-sleep routine 3. Extinction of inappropriate behavior at bedtime 4. Reinforcement of appropriate behavior for sleep
Session 3	1. Review of the sleep diaries 2. Difficulties encountered in implementing the guidance provided 3. Reinforce the importance of parental consistency in the application of extinction and reinforcement
Session 4	1. Review of the sleep diaries 2. Difficulties encountered in implementing the guidance provided 3. Reinforce the importance of parental consistency even when there is improvement in behavior
Session 5	1. Review of the sleep diaries 2. Reinforce the importance of parental consistency even when there is improvement in behavior 3. Feedback resulting from the intervention 4. Finalization

During the sessions, mothers received guidance on the bedtime routine and positive reinforcement as well as the use of the extinction techniques to improve sleep times and reduce the child’s nighttime awakenings. The respective guidance is described below:

Establishment of a bedtime routine and positive reinforcement: The mothers were instructed to use the positive reinforcement technique to teach the appropriate sleep behavior to their children. The establishment of pre-bedtime routines should take place by utilizing behavior that indicate bedtime, such as brushing teeth, putting on pajamas, going to the bedroom, lying down, listening to a story, and relaxing. During the execution of the technique, the mother was instructed to always reinforce—by giving attention and praise—when the children behaved appropriately, that is, those behaviors that favor the process of falling asleep, like staying calm, not crying, and staying in bed during the pre-sleep routine and immediately before going to sleep. Likewise, the caregivers were instructed to be careful not to reinforce the children’s inappropriate behaviors (crying, protesting).

##### Extinction

The extinction technique used in the present study was graduated extinction. Mothers were instructed, after completing the pre-sleep routine with the children, to ignore the children’s inappropriate behavior, that is, the behaviors that compete with that which is necessary to accommodate relaxation. In this variation of the technique, protests should be ignored for specific periods, i.e., in the first nights for five minutes, after a few nights for 10 min, gradually increasing the interval of the checks over the course of days. During the checks, the guidance provided was to reduce interactions with the children in order to avoid reinforcing the inappropriate behavior. At this point, caregivers were instructed to provide brief verbal responses (“I’m here, go to sleep”) if necessary, so that the checks lasted an average of 15 to 60 s.

The guidance provided was that the steps of gradual extinction were to be performed mainly at bedtime. The concept is that the generalization of the positive association for sleep onset remains during waking. In cases where the child awakened at night and sought the parents to sleep by sharing a bed, the advice was to guide the child back to his/her bed.

The guidelines were also provided on printed paper for the mothers to take home. In the space of time between sessions, the researcher would be available via telephone if the parents wished to call.

##### Waiting List

During the waiting period, the participants in the control group entered the notes in the sleep diaries with the use of actigraphs. The researcher contacted these participants weekly via telephone, questioning them about the sleep diaries entries.

All participants completed measures at pre-treatment, post-treatment, and at 1- and 6-month follow-up. After the reevaluation at the 1-month follow-up, the control group participants were provided the intervention and, once the intervention was completed, they were reevaluated again.

The measurement instruments used are described below.

### Instruments

#### Socio-demographic information

In the pre-treatment evaluation, the following socio-demographic information was collected: age, gender, date of birth, medical and psychiatric history of the child and age, level of education, marital status, and social stratum (ABEP, 2012) of the parents.

Composite Sleep Disturbance Score (CSDS): This measurement, adapted from the study by Richman and Graham (1971), gathers and organizes the data provided by the instruments taken from UNESP Scale of Sleep Habits and Hygiene—Child Version (Pires et al., 2012) and Sleep Disorder Scale for Children and Adolescents (Bruni et al., 1996) in order to contemplate the main variables of infant sleep and obtain a total score. The index includes the variables “resisting going to bed,” “sleep latency,” “night awakenings,” and “sleeping with parents,” providing a score ranging from 0 to 12, so that the higher the score, the greater the sleeping problem is. The data are collected on the pre- and post-intervention and follow-up assessment.

#### Sleep diary

Sleep logs were designed for parents to record the times the children slept and woke up, an estimate of how long it took to fall asleep and the number of awakenings during the night. The data from the sleep diary are collected daily. Actigraphy: the actigraph, a motor activity monitor, is an instrument equipped with an accelerometer. It has the shape and size of a watch, is used on the wrist, and is intended to indirectly measure sleep through the quantification and analysis of motor activity, while providing the following measurements: (1) sleep onset latency (time to fall asleep), (2) sleep efficiency (effective sleep time during total bed time, which is calculated as a percentage), (3) wake after sleep onset (awake time after awakening in minutes), and 4) total sleep time (Souza et al., 2003). The model used was AW-64 (Mini-Mitter Co., Inc.), and the records were analyzed using specialized software (Actiware-Sleep, v. 5.0). For the actigraphy analysis, only participants who had records for at least five nights at each stage (pre- and post-intervention and follow-up assessment) were considered. Thus, participants with incomplete records and absence of records in the pre, post or follow-up stages were excluded. The records were analyzed with a large threshold (= 80) to identify awakenings, which are more appropriate for children at this age. The device was used only at night on the non-dominant wrist.

#### Child Behavior Checklist 1½–5 years (CBCL)

This is an instrument developed by Achenbach and Rescorla (2000) to obtain standardized rates of behavioral problems in children based on their parents’ reports. The Brazilian version of the CBCL/ 1.5–5 presents good reliability indexes, evaluated in a non-probabilistic sample of 157 mothers. The test-retest analysis resulted in high intraclass correlation coefficients in the scales for internalizing (0.99), externalizing (0.99), and total problems (0.98). The internal consistency values (Cronbach’s alpha) range from 0.69 (somatic problems) to 0.94 (total problems) (Pires et al., 2014). By analyzing the items of these syndromes, a characterization of the child’s global functioning (clinical and non-clinical) is also obtained as well as the internalizing and externalizing profiles. For this study, the total of behavior problems was considered to be externalizing problems and internalizing problems. The data are collected on the pre- and post-intervention and follow-up assessments.

#### Adult Self-Report (ASR) (Achenbach & Rescorla, 2003)

ASR is a self-administered questionnaire for individuals from 18 to 59 years of age, comprising 126 items in total. This instrument evaluates aspects of the participants’ adaptive and psychopathological functioning. Each item has three response options that vary from one “not true” to two “very true or often true.” This instrument was translated into Brazilian Portuguese by (Rocha et al.: Validation evidences for the Brazilian versions of Adult Self Report (ASR) and Adult Behavior Checklist (ABCL), unpublished) and was shown to be reliable, having an internal consistency (Cronbach’s alpha) with a value above 0.6. In the present study, the alpha values for the different syndromes ranged from 0.6 to 0.8. For this study, the mother’s behavioral problems total score was used only as a covariate in the model for control, not as a measure of interest.

#### Satisfaction to treatment

Developed by the researchers, this scale includes four questions with possibilities of yes or no answers: “Has there been improvement in your child’s sleep problems?” “Has there been improvement in your child’s behavior problems?” “Was it difficult for you to apply this intervention to your child?” “Were you satisfied and would you recommend this intervention?” These questions were asked to mothers in the post-treatment phase.

### Outcome measures

The first hypothesis was that the behavioral intervention resulted in improvement of sleep in children, characterized by reduction in the sleep latency, in the night awakenings, in the behavior of resisting going to bed, and desire to sleep with their parents. These standards were assessed by way of an overall score of the CSDS, with the overall score of this instrument being the primary outcome. Other instruments were used to evaluate childhood sleep, such as the sleep diary and actigraphy. The results of these measurements were considered as a secondary outcome.

The second hypothesis was that the intervention could also result in improved daytime behavior by the children. Daytime behaviors were accessed through CBCL subscale scores: total behavioral problems, externalizing behaviors, and internalizing behaviors. These measurements were also considered to be secondary outcomes.

### Statistical analyses

Analyses were performed in SPSS 20.0 and a significance level of 0.05 was accepted for all comparisons, with Sidak-Bonferroni corrections. Descriptive analyses are presented as proportions, means, and standard deviation. For the continuous variables, comparisons were made through the *t* test for independent samples; for the ordinals, the *Z* test was used.

Means for the sleep diaries variables (sleep onset latency, number of night wakings, and total sleep time) were computed weekly for the whole protocol period (baseline; sessions 1, 2, 3, 4, and 5 of the intervention period; post-intervention; 1-month follow-up; and at 6-month follow-up). After the evaluation in the 1-month follow-up, there was a 1-week interval before the beginning of the intervention in the waiting list control group.

In the first phase, sleep assessments and other ways the children behave, measured by way of inventories, questionnaires, diaries, and actigraphy, were compared using a repeated measures ANOVA (Group Factor: intervention and control; Time Factor: pre-treatment, post-treatment, 1 month of follow-up, 6 months of follow-up. Six months of follow-up, only for the treatment group). As there were sibling children, the mother was placed as a random variable. In addition to the randomization effect, the model was composed of the main effects of the group factors; time of treatment, in addition to their interaction and the covariates; social stratum (criterion Brazil); and prior mental health (ASR-total) of the mother. The covariance structure for repeated measurements chosen was unstructured.

For the primary and secondary outcomes, the magnitude of the effect was calculated. The size of the effect was calculated at the time of the post-treatment and using Cohen’s *d*, calculated as suggested by Feingold (2009). The index was calculated based on the differences between the groups for the estimated means for each variable, divided by the standard deviation at baseline. Conventionally, a value of *d* = 0.20 represents a magnitude with a small effect, *d* = 0.50 indicates a magnitude with a medium effect, and d = 0.80 indicates a magnitude with a high effect (Cohen, 1988).

In the second phase, analyses were performed considering the data from the control group after it was submitted to intervention. Comparisons between the two moments (pre- and post-intervention) were performed by applying the *t* test and by calculating the magnitude of the effect.

## Results

**Fig. 1:**
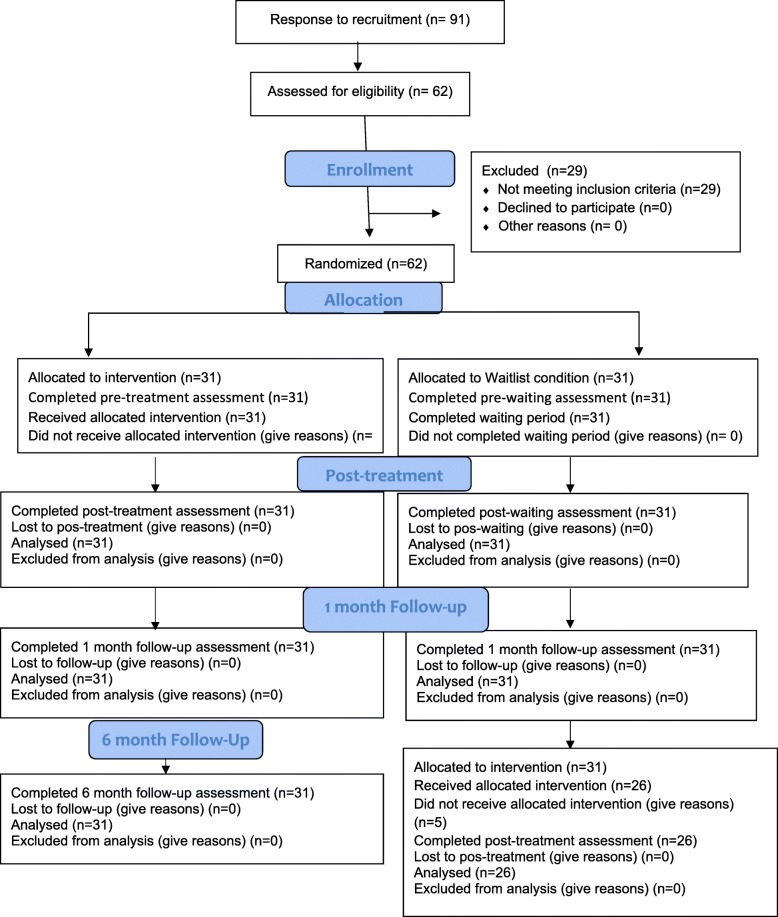
CONSORT Flowchart Showing the Progression of Participants Throughout the Study

A total of 62 participants met the eligibility criteria and were randomized into the control (waiting list) and intervention groups, as shown in Fig. 1. Each group consisted of 31 participants, all of whom performed pre- and post-intervention and follow-up assessments. At the time in which the intervention was offered to the control group, five mothers from this group declined to participate in the study because they could not attend the in-person sessions. The remaining 26 participants in the control group were submitted to the same behavioral intervention offered to the other group and then reassessed.

All the participants had an attendance of at least 80% when submitted to the intervention, which corresponds to four sessions and only one absence.

### Baseline clinical and socio-demographic characteristics of the participants

No differences were observed among the baseline characteristics of the two groups in terms of age (children and mothers), gender of the child, marital status of the mothers, and maternal psychopathology, presented in Table 2. However, mothers in the intervention group had a higher level of education and economic level, compared to the mothers in the other group.

**Table 2:** Socio-demographic characteristics of the participants

Characteristics of the child	Control g. *N* = 31	Intervention g. *N* = 31	Z	Total *N* = 62
Mean age (SD)	2.1 (1.2)	2.5 (1.4)	1.20 *Z*	2.3 (1.3)
Age, *N* (%)			Z = Z test	
12–42 months (1–3½ years), *N* (%)	23 (74%)	22 (71%)	0.28	45 (73%)
43–60 months (3½–5 years), *N* (%)	08 (26%)	09 (29%)	0.28	17 (27%)
Gender, *N* (%)				
Male	16 (52%)	18 (58%)	0.51	34 (55%)
Female	15 (48%)	13 (42%)	0.51	28 (45%)
Characteristics of the mother	Control g. *N* = 28	Intervention g. *N* = 26	Z	Total *N* = 54
Mean age (SD)	34.42 (6.0)	32.15 (5.2)	1.47 *Z*	33.3 (5.7)
Age, *N* (%)			Z = Z test	
20–29 years, *N* (%)	7 (25%)	7 (27%)	0.16	14 (26%)
30–39 years, *N* (%)	16 (57%)	17 (65%)	0.62	33 (61%)
40–49 years, *N* (%)	5 (18%)	2 (8%)	1.11	7 (13%)
Marital status				
Single	1 (3%)	2 (8%)	0.6	3 (6%)
Married	24 (86%)	21 (81%)	0.48	45 (83%)
Separated	3 (11%)	3 (11%)	0.09	6 (11%)
Education, *N* (%)				
Higher edu.	14 (50%)	20 (77%)	2.04*	34 (63%)
High school	11 (39%)	6 (23%)	1.28	17 (31%)
Elementary	3 (11%)	–	1.71	3 (6%)
Social stratum, *N* (%)				
High	5 (18%)	14 (54%)	2.76*	19 (35%)
Medium	18 (64%)	11 (42%)	1.61	29 (54%)
Low	5 (18%)	1 (4%)	1.63	6 (11%)
Maternal mental health				
Total problem ASR	58.0 (8.6)	58.7 (8.4)	0.30	58.35 (8.5)

*p < 0.05

*ASR* Adult Self Report

No differences were observed among the baseline characteristics of the two groups extracted from the sleep diary and questionnaires. For the variables of sleep onset latency (*t* = 2.38, *p* = 0.02) and the total sleep time (*t* = 2.03, *p* = 0.04) as measured by the sleep diary, after Bonferroni correction for multiple comparisons (*p* < 0.0125), *p* values were still without statistical significance.

### Change in primary and secondary outcomes in the first phase of the protocol

Means, standard deviations, and Cohen’s *d* were used for primary and secondary outcome measures in both groups in the first phase of the protocol and are displayed in Table 3.

**Table 3:** Primary and secondary outcome measures in the first phase of the protocol

	Pre-treatment		Post-treatment		Follow-up 1 month		Follow-up 6 month	Group (control and intervention)* Time (pre- and post-intervention and follow-up)		
	Control	Intervention	Control	Intervention	Control	Intervention	Intervention			
	Mean (SD) *N* = 31	Mean (SD) *N* = 31	Mean (SD) *N* = 31	Mean (SD) *N* = 31	Mean (SD) *N* = 31	Mean (SD) *N* = 31	Mean (SD) *N* = 31	F	P	Cohen’s *d* (post)
Sleep questionnaires										
CSDS	10.1 (1.6)	9.8 (2.0)	9.9 (2.3)	2.2 (2.2)	10 (2.3)	2.2 (2.3)	2.1 (2.4)	103.64	.000	3.83 (2.96–4.71)
Sleep diary										
Sleep latency (min)	58.9 (32.4)	40.8 (27.0)	59.8 (31.3)	18.5 (14.2)	59.7 (34.5)	18.2 (13.4)	17.3 (10.4)	9.25	.000	1.53 (0.94–2.12)
Number of night wakings	2.7 (1.7)	2.2 (1.2)	2.7 (1.5)	0.1 (0.3)	2.4 (1.6)	0.1 (0.2)	0.1 (0.2)	32.15	.000	0.64 (0.11–1.17)
Total sleep time (h)	9.2 (1.3)	9.8 (1.0)	9.3 (1.1)	10.3 (1.2)	9.3 (1.3)	10.1 (1.2)	10.2 (1.2)	1.32	.274	0.91 (0.37–1.46)
Daytime functioning questionnaires										
Total Problem CBCL	62.3 (10.2)	62.7 (9.5)	61.8 (10.9)	52.6 (8.7)	62.8 (11)	52 (8.9)	50.5 (9.6)	22.71	.000	0.59 (0.06–1.12)
Internalizing Problems CBCL	57.7 (10.4)	59.4 (10)	57.7 (10.8)	51 (10.1)	58.9 (10.8)	49.8 (10.7)	49.4 (10.5)	20.86	.000	0.37 (−0.14–0.89)
Externalizing Problems CBCL	60.1 (11.3)	59.7 (10)	59.6 (11.6)	52.8 (8.3)	60.5 (11.8)	52.6 (8.8)	50.5 (9.2)	10.17	.000	0.29 (−0.22–0.81)
	Mean (SD) *N* = 7	Mean (SD) *N* = 13	Mean (SD) *N* = 7	Mean (SD) *N* = 13	Mean (SD) *N* = 7	Mean (SD) *N* = 13	Mean (SD) *N* = 13	F	P	Cohen’s *d* (post)
Actigraphy										
Sleep latency (min)	3.0 (1.4)	3.5 (1.2)	4.9 (2.2)	2.2 (0.8)	3.1 (1.8)	2.4 (1.6)	3.3 (1.7)	10.68	.001	2.30 (1.04–3.57)
Sleep efficiency (%)	83.6 (5.3)	86.4 (3.9)	85.6 (3.6)	86.8 (4.8)	84.6 (5,0)	87.6 (4.8)	90.9 (2.8)	0.51		–
Wake after sleep onset (min)	84.8 (30.4)	74.3 (25.6)	69.0 (23.0)	69.9 (27.5)	71.9 (23.9)	66.1 (27.5)	46.2 (16.8)	0.82		–
Total sleep time (min)	454.6 (59.6)	514.1(25.8)	462.1 (49.2)	492.1 (50.3)	436.3 (55.2)	504.2 (43.1)	523.4 (33.9)	0.52		–

*CSDS* Composite Sleep Disturbance Score, *CBCL* Child Behavior Checklist

*=versus

There is a reduction in the scores of the primary and secondary measurements after the group was submitted to intervention, indicating improvements not only in the children’s sleep patterns, but also in daytime behavior. After treatment, the intervention group presented a significant reduction in the Composite Sleep Disturbance Score, with a large effect size, indicating the positive effect of the intervention on the improvement of the children’s sleep, characterized in this instrument by resistance in going to bed, sleeping with the parents, difficulty to fall asleep, and night wakings. The data also indicated average to high Cohen’s *d* values in the sleep diaries’ variables. The effect of the intervention was also positive in terms of the children’s daytime behavior, resulting in an improvement in total behavioral problems, with a medium Cohen’s value, due to an improvement in the problems of externalizing and internalizing behaviors.

Part of the sample had sleep evaluation by actigraphy with considerable frequency for the study. Compared to the control group, the intervention group had a statistically significant reduction in sleep onset latency after treatment with Cohen’s high value *d*. There were no detectable differences in the other variables.

**Fig. 2:**
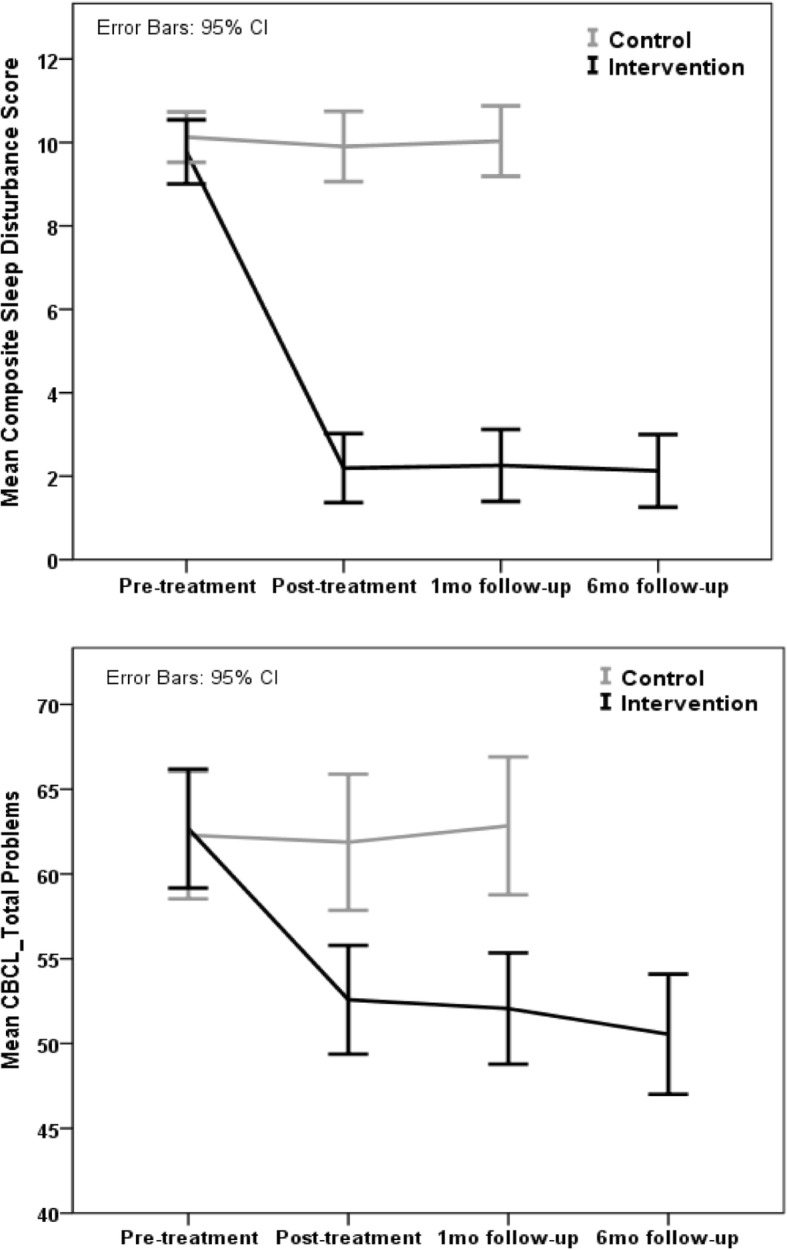
Difference between the groups in terms of the CSDS scores and total CBCL

Overall, we observed that the results were maintained in the follow-up period, while the control group remained stable over time. The trajectories of each group could be seen over time in Fig. 2 in relation to the CSDS variables and total CBCL.

### Change in primary and secondary outcomes in the second phase of the protocol

The results in the second phase were in line with the first phase analysis, indicating that the intervention resulted in improvements in the children’s sleep and daytime behavior, as assessed by the instruments, with high Cohen’s *d* values, as shown in Table 4.

**Table 4:** Primary and secondary outcome measures in the second phase of the protocol

Measures	Pre-treatment	Post-treatment	*t*	*d*
	Mean (SD) *N* = 26	Mean (SD) *N* = 26		
Sleep questionnaires				
CSDS	10.27 (1.6)	1.80 (2.6)	16.23*	3.91
Sleep diary				
Sleep latency (min)	50.14 (31.84)	18.18 (18.04)	6.59*	1.23
Number of night wakings	2.38 (1.31)	0.17 (0.46)	12.01*	2.25
Total sleep time (h)	9.54 (1.22)	10.22 (1.08)	3.15*	0.60
Daytime Functioning Questionnaires				
Total problem CBCL	61.19 (10.72)	49.42 (11.69)	6.52*	1.04
Internalizing problems CBCL	57.34 (10.65)	49.30 (11.31)	4.72*	0.73
Externalizing problems CBCL	58.73 (11.0)	48.96 (10.37)	6.06*	0.91

*p < 0,05

*CSDS* Composite Sleep Disturbance Score, *CBCL* Child Behavior Checklist

Regarding the results assessed by actigraphy, only one participant recorded complete data. The sleep quality of the child before the intervention was considered good, with a short sleep onset latency, a sleep efficiency greater than 85%, and a total nighttime sleep of more than 8 h. Despite this, the sleep-onset latency was reduced after the intervention.

These data reinforce the effect of the intervention, considering that when the control group was on the waiting list, it did not obtain improvement in the sleep patterns nor in the daytime behavior and only after being exposed to the intervention did it present the respective improvements.

### Satisfaction to treatment

Table 5 presents the proportion of mothers regarding their satisfaction to treatment.

**Table 5:** Satisfaction to treatment

	Total (*n* = 57)	
	*n*	%
Improvement in sleep problems	55	96
Improvement in behavior problems	54	95
Satisfaction/recommend treatment	57	100
Difficulties in applying	29	51

Most of the mothers reported improvement in sleep and behavior problems in their children. All of them reported satisfaction with the intervention and that they would recommend it to their friends. About half reported some difficulties while implementing the intervention with the child at home. No difference in satisfaction effects were observed between those who completed the intervention in Phase 1 or Phase 2.

## Discussion

This study was the first randomized controlled trial to evaluate the efficacy of behavioral intervention, based on a program aimed at parents, for childhood sleep problems in the Brazilian population. The advantages of this study include documenting the effect of intervention not only on sleep, but also on daytime behavior. The results of this study support our initial hypotheses. The intervention group was found to be superior to the wait list group in reducing bedtime problems, sleep onset latency, night wakings, and co-sleeping with parents, reported daily in the sleep diaries and in the interview, as demonstrated in the significant group by time interaction effect. The improvement in the child’s sleep was also verified by an objective measurement, which presented a reduction in sleep onset latency after the intervention was completed. The present results are in line with previous findings that attest to the effectiveness of behavioral interventions in childhood insomnia (Meltzer & Mindell, 2014; Mindell et al., 2006; Mindell et al., 2009; Mindell et al., 2011a, 2011b; Morgenthaler et al., 2006; Paine & Gradisar, 2011) but, again, this was the first study conducted in Brazil.

Compared to the control group, there was a significant reduction in the scores on all the measures that assessed sleep problems (CSDS, sleep onset latency, nocturnal awakenings) and behavioral problems (total CBCL, externalizing and internalizing problems CBCL). Moreover, the results were maintained at the follow-up. Importantly, analyses showed improvement in all variables after the control group underwent intervention. These results were confirmed on the maternal perception obtained following the intervention, with most mothers reporting high levels of satisfaction and improvement in their children’s sleep. These data show the positive effects of intervention not only on sleep but also on daytime behavior.

The results of the present study demonstrated significant effect sizes, in particular in regards to the CSDS measurements, while being in line with the results of previous research that indicated that children who had a behavioral intervention which included components intended to promote the child’s ability to self-calm and fall asleep independently, such as extinction for example, led to significant improvements in sleep quality, such as reduction in bedtime resistance (Adams & Rickert, 1989; Moore, Friman, Fruzzetti, & Macaleese, 2007), sleep latency (Blunden, 2011) and night wakings (Blunden, 2011; Moore et al., 2007; Seymour, Bayfield, Brock, & During, 1983). In the present study, extinction may have played a key role in reducing awakenings and the increased independence of the children at bedtime. By reducing parental involvement just before bedtime, children had the opportunity to develop self-regulating skills that are important for learning to sleep independently. According to Thomas et al. (2014), the empirical support and success in the results of the extinction technique is precisely because it achieves the objective of promoting the development of self-accommodation abilities in the child. In fact, the Allen, Howlett, Coulombe, and Corkum (2016) review strongly supports the recommendations that encourage independence at bedtime in order to break down negative associations with early onset of sleep.

This study demonstrated that in addition to improving the quality of sleep in children, the results also showed an improvement in the children’s behavior. Evidence of the effect size medium in terms of total behavioral problems of the CBCL scale was shown, indicating a significant effect of the intervention on behavior in general. The behavior problems of the control group did not improve while they were on the waiting list, which may indicate that they did not improve as a result of the passage of time. However, after being submitted to the intervention, the results were satisfactory. The second phase analysis revealed a high *d* value, indicating that the scores between the periods had a considerable difference. The possible association between sleep problems and behavior problems elucidates the importance of the treatment for childhood insomnia, showing a contribution to the improvement of the daytime behavior of the children.

Several studies have already indicated an association between sleep problems and problems in the internalizing and/or externalizing profile (Blunden & Chervin, 2008; Gregory et al., 2008; Hall et al., 2007; Rafihi-Ferreira et al., 2016; Scher et al., 2005; Sourander, 2001; Shang, Gau, & Soong, 2006; Stein et al., 2001; Storch et al., 2008). This was demonstrated in the results of the present study, corroborating with previous studies with a randomized design. Pinilla and Birch (1993) assessed the child’s temperament using the Bate’s Infant Characteristics Questionnaire, and, in comparison with the control group, the results for sleep problems showed that after the intervention, the parents reported that their children’s behavior was more predictable, to the extent that parents had greater control over their children. Seymour, Brock, During, and Poole (1989) found that 73% of parents reported positive changes in their children’s daytime behavior after undergoing behavioral therapy for insomnia.

For Mindell et al. (2006), a likely factor for this change is the increase in the amount and quality of sleep that children and their parents experience after the effective treatment. Another factor that is in need of evidence is that the intervention involves modifications of parents’ behaviors and the environment and consequently interferes with the children’s responses. The consistency of the parents’ behavior at bedtime, through setting boundaries, modifying the environment, and through routines, can also be generalized for daytime behavior. One hypothesis is that the parents started to establish routines and limits in the daytime period also, which may have contributed to behavioral improvement. Moreover, with the intervention, parents understand that their behavior often affects their children’s sleep problems and thus are instructed to modify such behavior. As changes occur, parents begin to handle the child’s problems differently, making them more independent. In this manner, in addition to improving sleep behavior and consequently sleep patterns, this factor may contribute to the improvement of the child’s daytime behavior.

Associations between sleep and behavior are complex and most likely bi-directional. This study corroborates previous studies that indicate an association between sleep problems and behavior problems, and that improvement in one can influence the other. The findings identified in this study emphasized the importance of sleep for daytime behavior, i.e., for emotional well-being, further stressing the importance of behavioral treatment for infant sleep. It is important to emphasize that in this study, these were typical children with no psychiatric problems. However, the role of emotional well-being in sleep quality is also evidenced, that is, whether or not factors related to children’s emotional well-being (e.g., mental health problems) appear to play a central role in maintaining or exacerbating sleep problems, for which referral and specialized treatment are important (Allen et al., 2016).

This study had certain limitations, such as the difficulty experienced by the participants when using the actigraphs, as only one third of the participants used actigraphy, thus compromising parts of the assessment of the quality of the sleep based on the objective measurement. In addition, the assessments of participants were not blinded and were conducted by the interventionist, which could introduce a bias into the results.

The durability of the treatment effects should be ascertained in future research, which would evaluate the maintenance of treatment results over a longer time-period. Research that assesses parental tolerance when confronted with the effects of extinction and which explores the factors that are associated with treatment success is necessary and may contribute to the development of resources that can meet the demands such as difficulties with treatment compliance.

Although the intervention was efficient for the variables analyzed, there was a 16% dropout rate in the control group before it was submitted to intervention. Statistical analysis (Mann-Whitney) did not indicate that these mothers are different from the others for the variables age, marital status, social stratum, educational level, and mental health. However, future studies may focus on the reasons for abandonment in order to improve general adherence to face-to-face treatment and to explore alternative possibilities such as distance treatment.

The present study, in consonance with previous studies, shows that behavioral interventions that include parental education on the child’s sleep, establishment of pre-sleep routines, extinction, and positive reinforcement are effective for sleep problems in childhood for the Brazilian population as well. This model of intervention, in addition to being effective, was also effective in a typical clinical practice context reported in the US study by Byars and Simon (2014), which pointed out the viability and clinical effectiveness of evidence-based treatment for childhood insomnia in an actual practice. Considering that the present intervention was applied in a clinical school, the results of this study support the idea that this treatment protocol can be applied in the contexts of clinical services, such as in the practice of a psychology clinic-school.

It is believed that the implementation of clinical training for the management of childhood insomnia in the psychology program will enable future professionals to deal with childhood sleep problems both in clinical practice and in other contexts that encompass a broad spectrum of healthcare programs.

## Conclusion

Based on the results of this study, it can be concluded that the behavioral intervention for childhood insomnia, through parental guidance, is effective in improving sleep quality and the daytime behavior of children in the infant and toddler age group. The importance of the environment is evidenced, along with the essential role of the parental relationship, the parent’s behavior in sleep patterns, and the behavior of children at bedtime.

Our findings that principles of behavioral analysis have an effective application in treating sleep-related problems, both nighttime and daytime symptoms and behaviors, reinforce the importance of sleep psychology as an area of expertise recently recognized in Brazil and certified by Brazilian sleep and psychological associations.

## Abbreviations

ASR: Adult Self-Report
CBCL: Child Behavior Checklist
CSDS: Composite Sleep Disturbance Score
